# Supplementary material for: Post-harvest treatment of wild-simulated ginseng under climate-smart environmental conditions
Source: PLoS One. 2025 Jun 18;20(6):e0326237. doi: 10.1371/journal.pone.0326237 (PMC12176193; doi:10.1371/journal.pone.0326237)
Supplement: S1 Appendix — (DOCX) [file pone.0326237.s001.docx]

Supporting Information

**Post-harvest Treatment of Wild-simulated Ginseng under Climate-smart Environmental Conditions**

Solhee Kim, Taegon Kim, Jungyeon Kim, Kyo Suh

**S1 Appendix.** **Cultivation techniques, environmental management, and container design.**

**Smart facility for post-treated WSG**

In this study, we utilized certified three-year-old wild-simulated ginseng (WSG) plants obtained from the Pyeongchang Wild Ginseng Association. A total of 440 specimens were carefully selected and divided into two experimental groups based on visual size assessment. Group A consisted of 220 roots identified as larger specimens, while Group B comprised 220 roots identified as smaller specimens. All specimens, including those weighing less than 1 g, were carefully weighed before transplantation.

For the cultivation containers, we modified 2 L plastic bottles by cutting them to a height of at least 20 cm and creating drainage holes at the bottom to ensure proper water management. The growth medium selected for post-harvest treatment was Premium Ginseng Soil (Shinsung Mineral Co., Ltd., Korea)—Korea’s first certified organic ginseng cultivation medium (Certification No. 1-2-109).

This specialized soil mixture was formulated with specific physicochemical properties optimized for ginseng cultivation. It maintained a moisture content of 40 to 60%, an organic matter content of 0.3 million Mg/m³, a pH between 5.3 and 6.5, and an electrical conductivity below 0.3 dS/m. Additionally, the growth medium contained precisely balanced nutrient levels, including vitamin B25, carotene 4, vitamin C 4, sodium 200, chromium 5, copper 150, nickel 100, and arsenic below 300. The primary components of peat moss and perlite were incorporated to ensure optimal soil structure and nutrient availability for ginseng root development over a 12-month effective period.

**WSG cultivation technique in smart facility**

Transplantation Technique

We transplanted three-year-old WSG on April 20, 2022, at an indoor facility in the central garden of Building 103 at Seoul National University’s Pyeongchang Campus, Gangwon Province (Fig. S1). The specimens were carefully transplanted into pots filled with the specialized soil mixture, maintaining vertical orientation to ensure optimal growth conditions. Each pot contained either one or two ginseng roots, arranged to ensure uniform light exposure. In cases where two roots were planted in a single pot, we paired specimens weighing less than 1 g with those weighing more than 1 g to account for potential growth rate differences. The planting configuration was carefully designed to optimize growing conditions for each specimen.

According to the technique described by Kim et al. (2019) [1], planting holes were dimensioned based on specific ratios based on the size of each ginseng root. The hole width was maintained at three to five times the root diameter, while the depth was set at two to three times the root length. These dimensions were critical for proper root development and stability. We covered the rhizome (brain-like portion of the root) with 3 to 5 cm of soil medium to protect it. These specifications were based on practical considerations: holes narrower than three times the root diameter were too tight for proper vertical insertion, while holes wider than five times the diameter failed to provide adequate support. Similarly, holes shallower than twice the root length risked excessive light exposure, whereas those deeper than three times the root length could impede proper growth due to over-burial.

Irrigation Technique

Water management was carefully controlled throughout the experiment, with specific attention to soil moisture content. Each specimen received between 1,900 mL to 2,100 mL of water weekly through a bottom-watering system, with the exact amount adjusted according to individual group conditions. This bottom-watering technique was initially chosen to ensure gentle and uniform water distribution without disturbing the soil structure.

After the WSG specimens had sprouted to a height of 5 cm or more, we confirmed that direct overhead watering could be implemented without causing soil erosion. Subsequently, both bottom-watering and spray irrigation methods were employed while closely monitoring growth conditions. Additionally, hypochlorous acid (HOCl) was used in irrigation to address concerns about ginseng’s vulnerability to bacterial infections in the soil. HOCl was chosen for its strong bactericidal properties and safe decomposition; it effectively destroys bacteria, and any excess naturally converts to water upon reacting with organic matter [2]. This approach was particularly relevant as ginseng is known to be susceptible to various soil-borne pathogens, and conventional wild-simulated cultivation typically lacks protective measures against microbial contamination.

Growth Environment Control Technique

Light intensity and temperature management are crucial factors in the post-harvest treatment of WSG. WSG has an optimal growth temperature range of 20 to 25°C and is relatively sensitive to high temperatures. The light saturation point varies considerably with temperature: at 15°C, ginseng can tolerate up to 30,000 lux, whereas at temperatures above 30°C, the light saturation point drops dramatically to below 5,000 lux. High temperatures can cause critical growth disorders, disrupting photosynthesis and increasing respiration, which inhibits root hypertrophy and reduces specific gravity.

For optimal post-harvest treatment, specimens were maintained in an environment where both light intensity and temperature were precisely controlled. While lower temperatures allowed for higher maximum light intensity thresholds, careful attention was paid to maintaining proper soil moisture through irrigation. The treatment environment was managed to maintain temperatures above 0°C after transplantation, with particular attention during summer months when temperatures exceeded 30°C. Light intensity was carefully controlled during these periods, not exceeding 200 PPFD (µmol/m²/s).

Temperature management focused particularly on soil conditions, with careful monitoring to ensure soil temperatures remained below 21°C. Additional irrigation was provided when necessary to maintain appropriate soil temperature and moisture levels. The treatment environment was explicitly selected for its naturally lower baseline light intensity, reducing the need for extra cooling or shading measures.

Light control was achieved using polyethylene shade nets installed below the upper treatment area, with natural light as the control condition. This shading system maintained light exposure within 5,000 lux, in line with the optimal growth requirements of WSG, and allowed for precise comparison between shaded and natural light conditions while protecting the specimens from excessive light exposure.

These conditions were selected based on the known physiological sensitivities of Panax ginseng. Elevated temperatures and high light intensity are known to impair photosynthetic efficiency and reduce biomass accumulation due to photoinhibition and increased respiration [4]. Prior studies have shown that ginseng exhibits optimal performance when light intensity is kept below 200 PPFD, particularly under cooler conditions [5, 6]. While humidity was not mechanically controlled, the shaded and enclosed indoor environment provided moderate and consistent humidity levels comparable to those in natural understory habitats.

**Light effect of WSG growth using Chlorophyll Fluorescence Measurement**

Chlorophyll Fluorescence Measurement

Light conditions are a critical factor affecting the growth of transplanted WSG. To evaluate the impact of different light conditions, specimens were cultivated for 15 d under natural light and shaded conditions using shade screens. The Fv/Fm value, which represents the maximum quantum yield of photochemical reactions in plant leaves, was used to assess photosynthetic activity [3]. It is calculated as follows:

Fv/Fm = (Fm - Fo) / Fm

where Fm is the maximum fluorescence after dark adaptation, Fo is the initial fluorescence after dark adaptation, and variable fluorescence (Fv) is the difference between Fm and Fo.

We selected healthy specimens for chlorophyll fluorescence measurements and measured the primary leaves from fully expanded compound leaves. Measurements were taken in duplicate for each specimen. Before measuring chlorophyll fluorescence, the leaves underwent a 30-minute dark adaptation period. Measurements were conducted using a Handy-PEA fluorimeter (Hansatech Instruments), and the data were processed using Handy PEA software version 1.31 to calculate the Fv/Fm values.

Changes in Photosynthetic Efficiency (Fv/Fm) According to Shading

While light is essential for photosynthesis, excessive exposure can damage the photosynthetic apparatus [4]. WSG, a shade-tolerant plant that grows optimally in partial shade and is sensitive to high temperatures, relies on relatively low light intensities for photosynthesis. Previous research has shown that leaf chlorophyll content decreases as light intensity increases in cultivated ginseng [5, 6].

We measured chlorophyll fluorescence in WSG leaves and calculated the Fv/Fm ratio to assess photosynthetic efficiency (Figure S1). The measurements were taken over 15 days, beginning 45 days after transplantation, under different light conditions. The results showed a general decline in Fv/Fm values across all treatment conditions over the observation period. Initial Fv/Fm values on day 1 were approximately 0.77–0.78, reaching their peak around day 5 at about 0.79–0.80 before gradually declining to 0.74–0.76 by day 15. However, statistical analysis revealed no significant differences in Fv/Fm values between the treatment conditions throughout the experimental period.

While the photosynthetic efficiency of WSG gradually decreased over time, the various light conditions tested in this study did not significantly impact the plants' photochemical efficiency. This non-efficiency indicates that the shading treatments provided adequate protection from photoinhibition while maintaining sufficient light levels for photosynthetic activity.

Effects of shading on shoot growth

The effect of light conditions on shoot development was evaluated by comparing leaf sizes between plants grown under natural light and those under shaded conditions. Leaf measurements were taken from the largest leaves on each stem for consistency. As shown in Figure S2A, plants under natural light developed notably larger leaves than those grown under shaded conditions, a trend consistent across different irrigation treatments.

Detailed morphological examination of the middle leaflets (Figure S2B) revealed that plants grown under natural light conditions (Light/H_2_O and Light/HOCl) exhibited larger leaf surface areas than those grown under shaded conditions (Dark/H_2_O and Dark/HOCl), suggesting that light availability was the primary factor influencing leaf development. These findings suggest that, although WSG is traditionally grown in heavy shade, it retains the capacity to develop larger photosynthetic surfaces when exposed to increased light exposure— an adaptation that must be carefully balanced with other physiological parameters to avoid light stress.

Effects of light intensity on root development

The relationship between light intensity and root development was evaluated by comparing the average weights of WSG roots grown under different light conditions. The experiment involved three-year-old or older wild-simulated ginseng roots with a mean initial weight of 1.70 ± 0.40 g (mean ± SD, n = 39). Measurements were taken two months after transplantation.

Figure S3 shows significant differences in root weight between plants grown under natural light and those under shaded conditions, while irrigation methods and water quantities were held constant. Plants under natural light conditions maintained an average root weight of approximately 3.5 g, whereas those under shaded conditions averaged about 2 g. This represents more than a 70% reduction in root weight under shaded conditions compared to those grown under natural light. These results demonstrate a strong positive correlation between light intensity and root growth in WSG, suggesting that adequate light exposure during post-harvest treatment is crucial for supporting root development and biomass accumulation.

References

1. Kim K, Jeong D, Kim H-J, Jeon K, Kim M, Um Y. A study on growth characteristics of wild-simulated ginseng (Panax ginseng CA Meyer) by direct seeding and transplanting. Korean Journal of Plant Resources. 2019;32(2):160-9. doi: <https://doi.org/10.7732/kjpr.2019.32.2.160>.

2. Kim C, Her Y, Kim Y, Jung C, Lim H, Suh K. Evaluating the effectiveness of HOCl application on odor reduction and earthworm population growth during vermicomposting of food waste employing. Plos One. 2019;14(12). doi: <https://doi.org/10.1371/journal.pone.0226229>.

3. Jung SY. Variation in antioxidant metabolism of young and mature leaves of subjected to drought. Plant Sci. 2004;166(2):459-66. doi: <https://doi.org/10.1016/j.plantsci.2003.10.012>.

4. Aro EM, Virgin I, Andersson B. Photoinhibition of Photosystem II. Inactivation, protein damage and turnover. Biochim Biophys Acta. 1993;1143(2):113-34. doi: <https://doi.org/10.1016/0005-2728(93)90134-2>.

5. Jang IB, Yu J, Kweon KB, Suh SJ. Effect of controlled light environment on the growth and ginsenoside content of Panax ginseng CA Meyer. Korean Journal of Medicinal Crop Science. 2016;24(4):277-83. doi: <https://doi.org/10.7783/KJMCS.2016.24.4.277>.

6. Song JN, Yang JL, Jeong BR. Growth and Photosynthetic Responses to Increased LED Light Intensity in Korean Ginseng (C.A. Meyer) Sprouts. Agronomy-Basel. 2023;13(9). doi: <https://doi.org/10.3390/agronomy13092375>.
