# Supplementary material for: Post-harvest treatment of wild-simulated ginseng under climate-smart environmental conditions
Source: PLoS One. 2025 Jun 18;20(6):e0326237. doi: 10.1371/journal.pone.0326237 (PMC12176193; doi:10.1371/journal.pone.0326237)
Supplement: S1 Fig — Light/H2O: natural light with water, Light/HOCl: natural light with HOCl, Dark/H2O: shaded with water, Dark/HOCl: shaded with HOCl. Measurements were taken at 1, 5, 8, 12, and 15 days after treatment. Vertical bars indicate standard errors. (DOCX) [file pone.0326237.s002.docx]

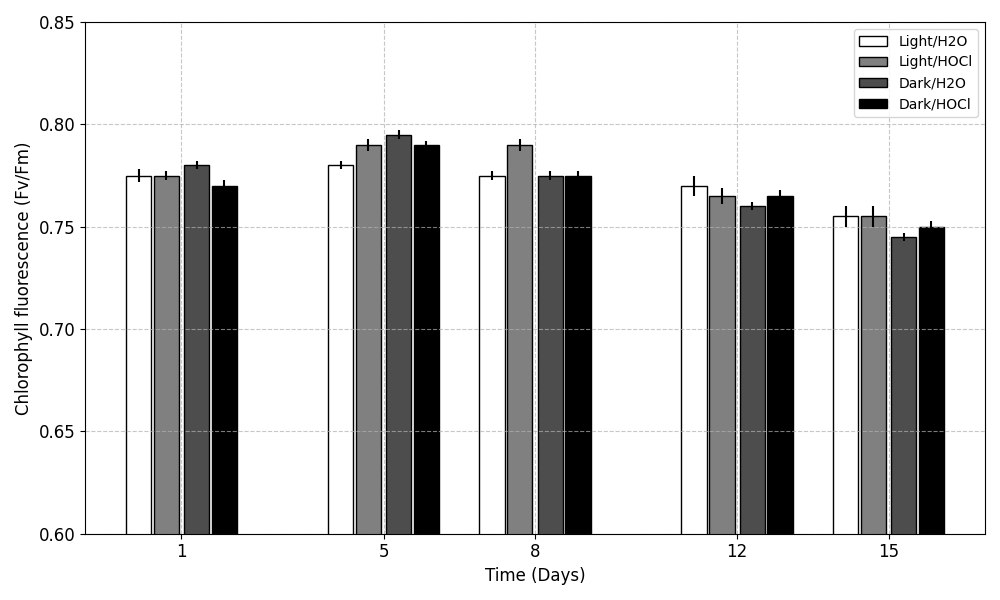


Figure S1. Changes in chlorophyll fluorescence (Fv/Fm) of WSG leaves under different light and irrigation conditions over a 15-day period. Light/H_2_O: natural light with water, Light/HOCl: natural light with HOCl, Dark/H_2_O: shaded with water, Dark/HOCl: shaded with HOCl. Measurements were taken at 1, 5, 8, 12, and 15 days after treatment. Vertical bars indicate standard errors.
