# Supplementary material for: Post-harvest treatment of wild-simulated ginseng under climate-smart environmental conditions
Source: PLoS One. 2025 Jun 18;20(6):e0326237. doi: 10.1371/journal.pone.0326237 (PMC12176193; doi:10.1371/journal.pone.0326237)
Supplement: S2 Fig — (A) Three petioles from plants grown under light and dark conditions. (B) Representative leaf morphology of middle leaflets under different light and irrigation treatments (Light/H2O, Light/HOCl, Dark/H2O, Dark/HOCl). Scale bar = 5 cm. (DOCX) [file pone.0326237.s003.docx]

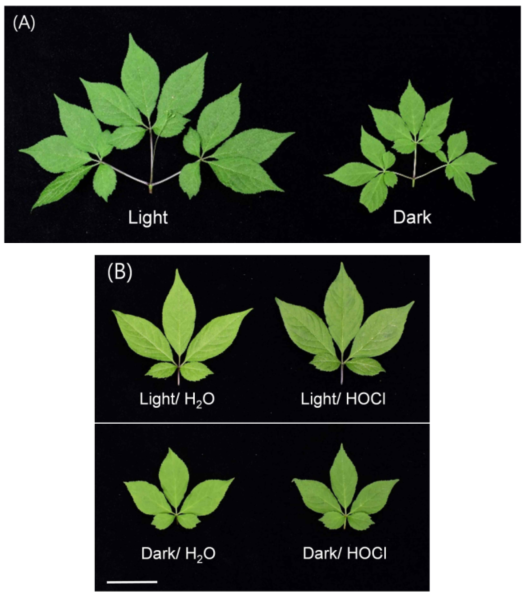


Figure S2. Comparison of shoot development under different light and irrigation conditions. (A) Three petioles from plants grown under light and dark conditions. (B) Representative leaf morphology of middle leaflets under different light and irrigation treatments (Light/H_2_O, Light/HOCl, Dark/H_2_O, Dark/HOCl). Scale bar = 5 cm.
