# Supplementary material for: Post-harvest treatment of wild-simulated ginseng under climate-smart environmental conditions
Source: PLoS One. 2025 Jun 18;20(6):e0326237. doi: 10.1371/journal.pone.0326237 (PMC12176193; doi:10.1371/journal.pone.0326237)
Supplement: S3 Fig — The initial weight of transplanted roots was 1.70 ± 0.40 g (mean ± SD, n = 39). Bars represent mean values ± standard errors (n.s.: not significant, ***: p < 0.001, ****: p < 0.0001). (DOCX) [file pone.0326237.s004.docx]

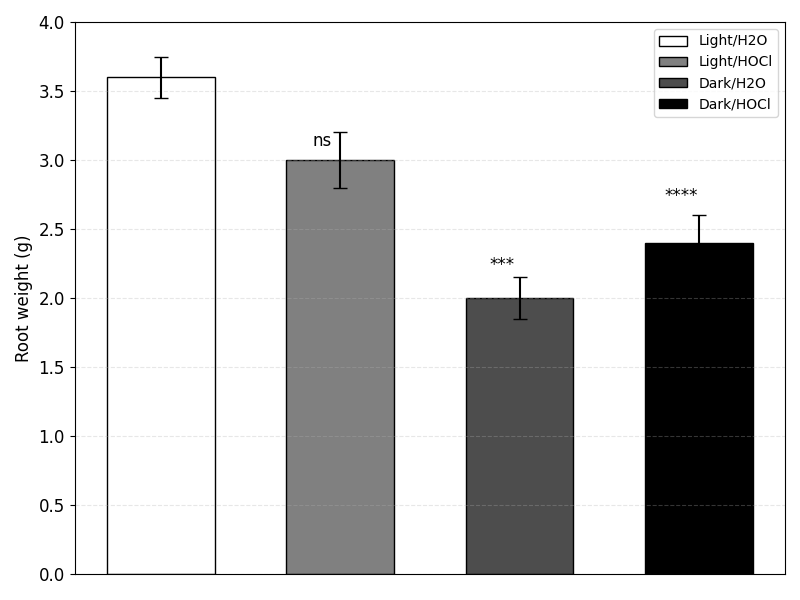


Figure S3. The average weight of WSG roots under different light and irrigation conditions after two months of treatment. The initial weight of transplanted roots was 1.70 ± 0.40 g (mean ± SD, n = 39). Bars represent mean values ± standard errors (n.s.: not significant, ***: p < 0.001, ****: p < 0.0001).
